# Addressing Oxidative Stress and Endothelial Dysfunction in Chronic Respiratory Diseases: The Role of Exercise and Multidisciplinary Rehabilitation

**DOI:** 10.3390/antiox13121543

**Published:** 2024-12-17

**Authors:** Pasquale Ambrosino, Maria Nolano, Claudio Candia, Guido Grassi, Mauro Maniscalco

**Affiliations:** 1 Istituti Clinici Scientifici Maugeri IRCCS, Scientific Directorate of Telese Terme Institute, 82037 Telese Terme, Italy; 2 Istituti Clinici Scientifici Maugeri IRCCS, Neurological Rehabilitation Unit of Telese Terme Institute, 82037 Telese Terme, Italy; maria.nolano@icsmaugeri.it; 3 Department of Neurosciences, Reproductive Sciences and Odontostomatology, Federico II University, 80131 Naples, Italy; 4 Department of Biomedicine, Neurosciences and Advanced Diagnostics, University of Palermo, 90127 Palermo, Italy; claudio.candia@unipa.it; 5 Department of Medicine and Surgery, Milano-Bicocca University, 20126 Milan, Italy; guido.grassi@unimib.it; 6 Istituti Clinici Scientifici Maugeri IRCCS, Pulmonary Rehabilitation Unit of Telese Terme Institute, 82037 Telese Terme, Italy; 7 Department of Clinical Medicine and Surgery, Federico II University, 80131 Naples, Italy

Chronic respiratory diseases (CRDs) are highly prevalent conditions, causing over 4 million deaths every year and representing a major source of physical, psychological, and occupational disability [1]. While the term CRDs is typically linked to common conditions like asthma and chronic obstructive pulmonary disease (COPD), the Global Burden of Disease Dictionary currently extends its definition to encompass a wide range of disorders with different etiologies that can affect any part of the airways [1]. Despite these varied etiologies (e.g., infectious, occupational, environmental, genetic), scientific evidence suggests that inflammation and oxidative stress may serve as common pathogenetic mechanisms in most CRDs, acting as key determinants of disease progression [2]. In this scenario, it is important to emphasize that oxidative stress and chronic inflammation are rarely confined to the lungs but are more often systemic, capable of disrupting vascular homeostasis [3]. In particular, oxidative stress is able to impact the vasculature in both pulmonary and peripheral circulation through a number of mechanisms, including peroxynitrite formation with reduced nitric oxide (NO) bioavailability, NO synthase uncoupling, accumulation of asymmetric dimethylarginine (ADMA), and increased arginase activity [3]. Thus, prompted in part by research on convalescent coronavirus disease 2019 (COVID-19) patients [4], the resulting endothelial dysfunction has increasingly been recognized as a key contributor to the systemic manifestations of CRDs and, as the earliest event in atherosclerosis, the associated cardiovascular risk [2].

It follows that monitoring endothelial function could have significant implications for the management of CRDs, with a variety of clinical and laboratory methods currently available [5]. Laboratory methods mainly focus on detecting molecules, membrane proteins, and mediators involved in the regulatory functions of the endothelium. In this scenario, it is important to emphasize that, due to the strong interconnection between inflammation, oxidative stress, and endothelial dysfunction, numerous biomarkers are shared across these processes, thus enabling comprehensive monitoring [3]. Among these, the most commonly used include (but are not limited to) peroxynitrite, asymmetric dimethylarginine (ADMA), vascular cell adhesion molecule-1 (VCAM-1), and malondialdehyde as indicators of both oxidative stress and endothelial dysfunction, alongside circulating endothelial cells (CECs), endothelial progenitor cells (EPCs), or endothelin-1 (ET-1), which are more specific to endothelial function [5]. Regarding clinical methods, both invasive and non-invasive techniques have been proposed over time, based on different principles, including venous occlusion plethysmography (VOP), laser Doppler flowmetry (LDF), and peripheral artery tonometry (PAT) [5]. However, one of the most widely used techniques for endothelial function assessment is the measurement of flow-mediated dilation (FMD) of the brachial artery, which has emerged as both an independent predictor of cardiovascular events and a surrogate marker of vascular oxidative stress [6].

Using these methods, several authors have monitored endothelial function in CRDs, and, supported by robust meta-analytic data [7], the disruption of endothelial homeostasis has earned recognition as a constant in CRDs and, therefore, as a novel and attractive therapeutic target [8]. However, there are currently no specific medications designed to treat isolated endothelial dysfunction, and its improvement still remains an unexpected but welcome side effect of various existing drugs [5], including renin–angiotensin system (RAS) inhibitors and statins, as well as some antioxidant and nutraceutical strategies [3]. Additionally, certain exercise-based approaches have shown the potential to mobilize EPCs, support mitochondrial function, activate sirtuins, and reduce inflammation and oxidative stress through the involvement of specific pathways, such as nuclear factor erythroid 2–related factor 2 (Nrf2) [9].

Since 1986, when the potential benefit of exercise on endothelial function in tennis players was first hypothesized [10], several studies have been published on this topic, with some promising and (sometimes) conflicting results also in regard to CRDs. A number of reports have highlighted the link between exercise capacity and endothelial function in CRDs, along with a consistent association between physical activity levels and the extent of endothelial dysfunction. In a pioneering paper by Barr et al. [11], it was first documented that there is a strong relationship between endothelial function measured by FMD and the severity of airflow obstruction across a heterogeneous group of former smokers. This result was later confirmed among COPD patients, along with the finding of a stronger association between forced expiratory volume in 1 s (FEV_1_) and endothelial function in physically less active patients [12]. Accordingly, with the awareness that strenuous exercise can conversely increase oxidative stress and inflammation [13], physical inactivity has been linked to increased vascular lipid peroxidation, upregulated superoxide release, and systemic inflammation in both animal models [14] and COPD patients [15], thereby determining endothelial dysfunction and accelerated atherosclerosis. Inactive COPD patients also show increased arterial stiffness, as indicated by higher values of pulse-wave velocity (PWV) [16]. This pairs well with the evidence that physical inactivity is one of the strongest predictors of overall and cardiovascular mortality in COPD [17]. Moreover, when assessing peripheral endothelial function through the reactive hyperemia index (RHI) measured by PAT, COPD patients with endothelial dysfunction (RHI ≤ 1.67) demonstrate poorer aerobic exercise capacity and a higher prevalence of cardiovascular risk factors [18]. A similar significant positive correlation between exercise capacity, measured by the 6-min walking distance, and endothelial function, assessed through FMD, can also be found among patients hospitalized for severe acute exacerbations of COPD [19].

The observation that physical activity levels and exercise capacity may somehow be linked to endothelial function in COPD has raised questions about whether exercise-based approaches could serve as effective strategies for reversing endothelial dysfunction in CRDs, thereby potentially reducing the associated cardiovascular risk. In a randomized controlled trial involving patients with COPD, 12-week exercise training led to a significant improvement in peak work rate, along with a reduction in blood fibrinogen, leptin, and late EPCs [20]. Despite some isolated conflicting findings [21], the latter result is substantially also confirmed when adopting FMD for endothelial function assessment and supervised training protocols with shorter durations (8 weeks) [22], or even by modest increases in daily step counts [23]. Accordingly, although PWV is not strictly considered a direct measure of endothelial dysfunction but rather an indicator of a consequential event, namely arterial stiffness, it is noteworthy that endurance exercise appears to improve this parameter as well [24].

Turning to a more structured approach in the context of a rehabilitation program, the scientific literature presents less consistent results, particularly highlighting some differences between measures of endothelial function and arterial stiffness. In patients with stable pulmonary arterial hypertension (PAH), rehabilitation appears to have a positive effect on endothelial function, promoting mechanisms that favor repair over damage, as evidenced by an increase in the number of CECs (CD34^+^/CD45^low^) and a reduction in endothelial microvesicles (CD31^+^/CD42b^−^) following training [25]. Similar findings are observed among stable group E COPD patients, in whom an almost 50% improvement in endothelium-dependent FMD can be expected at the end of an in-hospital rehabilitation program [26]. This improvement appears to be independent of major clinical and demographic variables, with one relevant exception, namely hypercholesterolemia. The well-known phenomenon of endothelial lipotoxicity seems to prevent hypercholesterolemic COPD patients from achieving favorable changes in endothelial function following rehabilitation [26]. Furthermore, scientific evidence suggests that improvements in endothelial function correlate with improvements in pulmonary functional parameters after completing the rehabilitation program [26]. Very similar results are also observed in less severe COPD patients [27] and even among convalescent COVID-19 patients accessing the rehabilitation setting within 3 months of testing negative [4]. On the other hand, while some authors report a similar improvement in arterial stiffness, correlated with favorable changes in blood pressure values [27], others fail to observe significant modifications in PWV and/or the augmentation index at the end of the rehabilitation program [28]. In an attempt to interpret these apparently conflicting results, it is important to first note that endothelial dysfunction is an early phenomenon of atherosclerosis and, by definition, reversible even in relatively short periods, whereas arterial stiffness is a consequential event associated with the accumulation of protein material (i.e., collagen) in the arterial wall, which reduces vessel distensibility. Therefore, according to some preliminary evidence [29,30], it is reasonable to assume that any improvement in arterial stiffness may require a longer intervention and/or depend on other concomitant factors, including physical activity levels or baseline exercise capacity.

To date, what we know for certain is that inflammation, oxidative stress, and accelerated atherosclerosis are closely interconnected processes in CRDs, with cardiovascular comorbidity accounting for a substantial proportion of the excess disease burden in these clinical settings. Current evidence is more easily interpreted when considering that, based on meta-analytical data, for each percentage point reduction in FMD, the risk of ischemic events increases by 12% [6], while a difference of ≥0.5 m/s in ankle-brachial PWV corresponds to a 7.5% reduction in cardiovascular risk [31]. Therefore, given that exercise-based strategies seem to positively impact these parameters, one could speculate that pulmonary rehabilitation may become the ideal healthcare setting for managing cardiovascular comorbidities in CRDs. However, several questions still remain unanswered, and further research is still needed. First, most of the current evidence on this topic concerns COPD, but other respiratory conditions, such as PAH or interstitial lung diseases, are endothelial cell disorders, so it is now necessary to determine whether the key role of endothelial dysfunction as a pathogenic mechanism and potential therapeutic target can be extended to pathologies other than COPD. Another issue concerns whether, within a multidisciplinary approach, additional factors beyond exercise (e.g., pharmacological adjustments) could contribute to the observed improvement in endothelial function and how to optimize their impact. Other aspects to explore pertain to whether different training modalities may have different effects on endothelial function, whether any improvements are temporary, and how long they persist after completing the rehabilitation program. Significant efforts are now required to better understand how (and to what extent) exercise and pulmonary rehabilitation may help restore endothelial function and manage CRDs along with their associated cardiovascular comorbidities.

## Data Availability

No datasets were generated or analyzed during the current study.
